# Perinatal Exercise and Cardiovascular Disease Risk

**DOI:** 10.1016/j.jacadv.2025.101776

**Published:** 2025-05-12

**Authors:** Marnie K. McLean, Bradley J. Petek, Lidija McGrath, Emily McGill, Abbi D. Lane

**Affiliations:** a Department of Applied Exercise Science, School of Kinesiology, University of Michigan, Ann Arbor, Michigan, USA; b Adult Congenital Heart Disease, Knight Cardiovascular Institute, Oregon Health and Science University, Portland, Oregon, USA; c University of South Carolina School of Medicine, Columbia, South Carolina, USA

**Keywords:** cardiovascular disease– physical activity, exercise, perinatal, pregnancy

## Abstract

The purpose of this narrative review was to summarize perinatal exercise guidelines and associations of perinatal physical activity and/or exercise with cardiovascular disease (CVD) risk. Observational studies, randomized controlled trials, systematic reviews, and meta-analyses were included. Gaps in literature and suggestions for future studies were identified. Despite concordant international guidelines, data to support nuanced activity advice for some subgroups are limited. Perinatal physical activity and exercise are consistently recommended to combat traditional CVD risk factors during the perinatal period, like excessive gestational weight gain, high blood pressure, and high blood glucose. Physical activity and exercise appear to improve nontraditional risk factors such as poor sleep and depression. Data are emerging regarding associations with some pregnancy-specific factors, such as placental characteristics. Further research investigating associations with pregnancy-specific CVD risk factors and associations in the longer term, as well as data to support uptake, adherence, and resistance exercise prescription is warranted.

## Highlights

• Perinatal exercise guidelines address programming, safety issues, and some pregnancy-related anatomical or physiological changes.
• Perinatal exercise can improve most traditional and some nontraditional cardiovascular disease risk factor determinants.
• Independent and joint associations of perinatal exercise with pregnancy/perinatal-specific factors like placentation and lactation should be investigated.
• Identifying optimal timing and dosing exercise strategies is important for improving perinatal and longer-term cardiovascular disease health.

**Figure 1:**
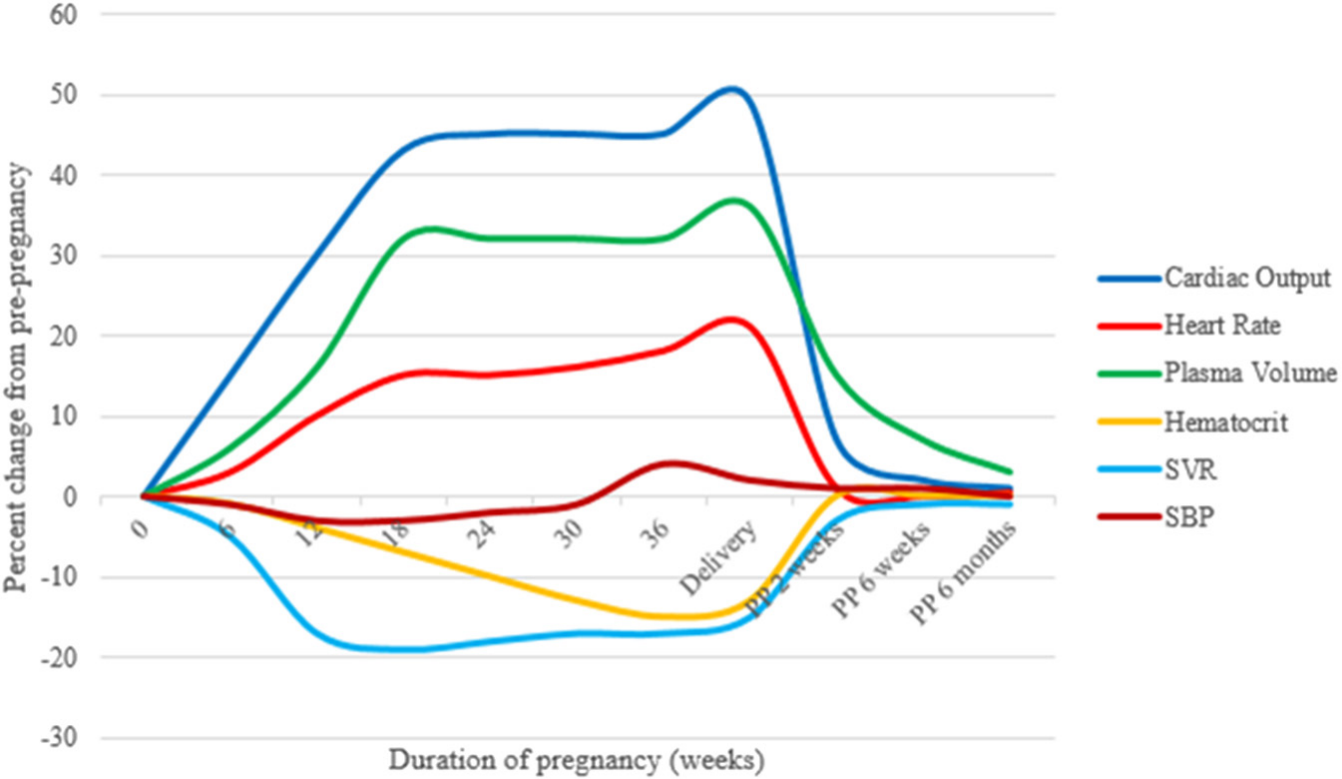
**Hemodynamic Adaptations to Pregnancy** Adapted from Yucel et al.^3^

Pregnancy is a cardiometabolic challenge.^1^ Even in uncomplicated pregnancies, individuals become more insulin resistant, and cholesterol levels increase.^2^ Hemodynamic adaptations occur: increased cardiac output, heart rate, plasma volume, and red blood cell mass, along with decreased systemic vascular resistance and hematocrit (Figure 1).^1^ Normotensive pregnancy is characterized by a small, transient fluctuations in systolic blood pressure (BP) (Figure 1).^3^ Impaired hemodynamic adaptations may represent unmasking of pre-existing subclinical cardiovascular dysfunction and/or an independent process that contributes to long-term complications.^3^ Compared to other developed nations, maternal morbidity and mortality rates in the United States are high and rising, reaching 23.8 deaths/100,000 live births in 2020.^4^ Cardiovascular conditions are the leading cause of maternal mortality, accounting for >25% of all pregnancy-related deaths.^5^ Reasons include more women entering pregnancy with risk factors, poor access to healthcare, and unfavorable social determinants of health.^4^^,^^5^

Evidence unequivocally demonstrates that regular exercise can prevent and/or treat adverse cardiometabolic conditions in many populations.^6^ Physical activity (PA) or exercise is associated with lower risk of novel and traditional cardiovascular disease (CVD) risk factors in the perinatal period,^6^ including adverse pregnancy outcomes (APOs) (hypertensive disorders of pregnancy [HDPs], gestational diabetes [GDMs], preterm birth, small-for-gestational-age deliveries).^7^^,^^8^ Offspring of individuals who exercise during pregnancy have better cardiovascular health, body composition, and nervous system development, with benefits extending into childhood.^9^

The goal of this narrative review is to synthesize literature related to perinatal PA/exercise, including national guidelines and effects of perinatal exercise on short- and long-term maternal CVD risk. This paper refers to PA as “any bodily movement resulting in energy expenditure,” while exercise is defined as “a subset of PA that is structured, repetitive, and aimed at maintaining or improving physical fitness.”^10^

## Methods

Studies published in English were identified in PubMed and Google Scholar databases. Search terms included “pregnancy,” “pregnant,” “postpartum,” “perinatal,” “cardiovascular disease,” “risk factors,” “blood pressure,” “glucose,” “lipids,” “cholesterol,” “obesity,” “BMI,” “smoking,” “arterial stiffness,” “endothelial function,” “autonomic function,” “Flt-1,” “sleep,” “diet,” “inflammation,” “placenta/l,” “mood,” “anxiety,” “depression,” “exercise,” and “physical activity.” Observational studies, randomized controlled trials, systematic reviews, and meta-analyses were screened. When consensus statements, systematic reviews, and/or meta-analyses were found, conclusions were included. Results from individual studies were included if they specifically addressed the risk factor of interest and were conducted in a large or specific population or were one of the few existing studies related to a particular risk factor.

### Perinatal exercise and PA recommendations worldwide

The benefits of exercise for reducing CVD risk are well described.^11^ Although benefits are clear, many individuals of reproductive age do not meet national PA guidelines including 150 min/wk or 500 MET·min/wk of moderate-vigorous aerobic activity and muscle-strengthening activities,^6^ and PA is frequently reduced during pregnancy and parenting.^12^

#### Current American College of Obstetrics and Gynecology guidelines

The American College of Obstetrics and Gynecology (ACOG) guidelines were updated in 2020 (Table 1).^13^ Previously, organizations recommended maintaining heart rates under specific levels (eg, 140 beats/min) due to wellbeing concerns.^15^ Guidelines now suggest using a subjective measure, the rating of perceived exertion (RPE), to monitor intensity as heart rate changes significantly during pregnancy.^13^ The RPE scale describes the individual's perception of physical work.^16^ Moderate-intensity exercise should elicit an RPE of 13 to 14 on the 6 to 20 scale, or “somewhat hard.”^13^^,^^16^ ACOG guidelines provide information about exercise frequency, type, intensity, and some information on duration.^13^ Musculoskeletal adaptations and injuries, as well as lower back, hip, leg, and foot pain are common^17^ and require consideration when prescribing perinatal exercise. Individualized or dynamic exercise prescriptions may be needed due to changes in body habitus and pre-existing or newly acquired comorbidities.^13^

**Table 1:** Detailed Summary of PA and Exercise Guidelines During Pregnancy and Postpartum

Exercise Principle	Recommendation	Red Flags
Frequency	150 min spread out throughout the week for aerobic exercise; no specific dosing in guideline statements for resistance exercise; nonconsecutive days are recommended for resistance training in nonpregnant populations	• Vaginal bleeding • Abdominal pain • Regular painful contractions • Amniotic fluid leakage • Dyspnea before exertion • Dizziness/lightheadedness • Headache • Chest pain, pressure, or tightness • Muscle weakness or fatigue affecting balance • Calf or extremity pain or swelling
Intensity	Moderate subjective exertion level of 12-14 on Borg Scale; <60%-80% of max effort; can say more than a few words or a sentence at a time during exercise but could not sing during exercise; Can continue vigorous activity if vigorous activity was performed prior to pregnancy; Moderate intensity resistance training characterized by >8 repetitions to volitional fatigue per set	
Time	Work up to 30-60 min/session; Can begin with shorter durations until >20-30 min is possible	
Type	Non-contact aerobic training free from fall risk; resistance training including bands, bodyweight, or weights; pelvic floor exercises	
Environment & safety	Thermoneutral and preferably supervised setting; ensure hydration; avoid lying on back during pregnancy; avoid Valsalva maneuver during resistance training; avoid hypoglycemia; empty breasts before exercising if lactating	
Timing	From first trimester until delivery as tolerated; may resume as soon as it is safe; consult a healthcare provider after complicated birth or c-section	

Adapted from ACOG Committee Opinion, Number 804, National PA Guidelines, and the 2023 AHA Resistance Training Scientific Statement.^6^^,^^13^^,^^14^ Exercise physiology principles can be applied to the perinatal population for exercise prescription. Special considerations and red flags contraindicating exercise or necessitating exercise termination are included. Pregnant people should consult with a health care provider before beginning exercise. Hypoglycemia can be avoided by limiting the intensity or duration (<45 min) of exercise sessions and/or by consuming adequate calories before exercise.

#### Current ACOG guideline gaps

While resistance exercise is recommended, specific exercise prescription information is lacking in ACOG guidelines.^13^ For discussion of ACOG guidelines for patient subgroups and other guideline gaps, please see the Supplemental Appendix.

#### Global guidelines

Many countries developed their own perinatal exercise recommendations.^18^ While generally concordant, guidelines do not always have comprehensive exercise prescription recommendations (Table 2). A 2017 review reported prenatal walking, stationary cycling, aerobic exercises, dancing, resistance exercises, stretching exercises, hydrotherapy, and water aerobics have all been extensively studied and were safe and beneficial.^19^ Authors concluded that all pregnant individuals without a contraindication should engage in regular PA and exercise.^19^ Meta-analyses reported that women of normal weight (n = 2,059) who engage in aerobic exercise experience 49% lower incidence of GDM (2.9% vs 5.6%; relative risk: 0.51; 95% CI: 0.31-0.82) and 79% lower incidence of HDPs (1.0% vs 5.6%; relative risk: 0.21; 95% CI: 0.09-0.45).^20^

**Table 2:** Summary of Perinatal PA and Exercise Guideline Components by Country

Country	Frequency Days/Week	Intensity	Duration		Type		Prior Activity Level		SB
			W	D	AE	RE	Inactive	Active	
Australia	✓	✓	✓		✓	✓	✓	✓	✓
Austria		✓	✓		✓	✓	✓	✓	✓
Belgium	✓	✓	✓		✓	✓		✓	✓
Brazil		✓	✓				✓		✓
Brunei	✓	✓	✓		✓	✓	✓	✓	
Canada	✓	✓	✓		✓	✓	✓	✓	
Chile	✓	✓		✓	✓	✓			
Cyprus	✓	✓		✓	✓				✓
Denmark	✓	✓		✓	✓	✓	✓		
Estonia	✓	✓	✓				✓		✓
Fiji	✓	✓		✓					✓
Finland	✓	✓	✓			✓	✓	✓	✓
France	✓	✓		✓	✓	✓	✓		
Greece	✓			✓			✓		
Iceland	✓	✓		✓	✓		✓		
Kenya		✓	✓		✓		✓		
Latvia	✓	✓							✓
Malaysia	✓	✓	✓			✓	✓		
NZ	✓	✓	✓		✓	✓		✓	✓
Norway		✓	✓				✓	✓	
Portugal	✓		✓					✓	
Qatar	✓	✓		✓	✓	✓	✓		✓
Singapore	✓	✓	✓	✓	✓	✓	✓		
Slovenia	✓	✓		✓		✓	✓		
Spain	✓	✓	✓			✓	✓	✓	✓
Sri Lanka	✓	✓	✓		✓				
Switzerland	✓	✓	✓			✓	✓		✓
UK		✓	✓			✓	✓		
USA	✓	✓	✓		✓	✓	✓		
Uruguay		✓	✓		✓	✓			✓

About half countries provide a weekly while half provide a daily exercise volume recommendation. Most countries provide frequency and intensity guidance. About half countries provide guidance on aerobic and resistance exercises. Most countries provide information regarding inactive people who become pregnant, with less information given for active women. Guidelines on sedentary behavior are provided in less than half the guidelines.

Adapted from Hayman et al.^18^

AE = aerobic exercise; D = daily; NZ = New Zealand; RE = resistance exercise; SB = sedentary behavior; UK = United Kingdom; USA = United States of America; W = weekly.

### Pregnancy PA or exercise and CVD risk factor determinants during pregnancy and after delivery

Studies evaluated associations of perinatal exercise/PA and CVD risk factors. Summaries according to individual risk factors are found below with general effects shown in Table 3.

**Table 3:** Perinatal Exercise and Traditional and Novel CVD Risk Factor Determinants

Risk Factor Determinant	Influence of Perinatal PA or Exercise
Traditional CVD risk factor determinants	
Blood pressure	↓ SBP, ↓DBP^21^
Glucose	↓ Fasting blood glucose, ↓ risk of GDM^22^
Obesity	↓ Risk of excessive GWG, ↓ postpartum weight retention^23^
Body composition	↑ Improved maternal and fetal body composition ↓ Maternal fat mass,^24^ ↓ fetal fat mass^25^
Lipid profile	↑ Improved maternal LDL and triglyceride levels^26^^,^^27^
Novel CVD risk factor determinants	
APOs	↓ Risk of APOs, PE, GDM, LBW, PTB^19^
Sleep	↑ Improved sleep quality^28^^,^^67^
Depression and anxiety	↓ Risk of PPD^13^
Cardiorespiratory fitness	↑ Fitness^13^ and ↓HDP^29^
Inflammation	↓ C-reactive protein^30^, ^31^, ^32^
Arterial stiffness	↔/? Pulse wave velocity^33^
Endothelial function	↑ Endothelial-dependent dilation^34^
Cardiac autonomic control	↓ Heart rate, ↑ HRV^35^^,^^36^
Lactation	↔ Breastmilk composition, volume, infant growth^37^

APO = adverse pregnancy outcomes; CVD = cardiovascular disease; DBP = diastolic blood pressure; GDM = gestational diabetes mellitus; GWG = gestational weight gain; HDP = hypertensive disorders of pregnancy; HRV = heart rate variability; LBW = low birth weight; LDL = low-density lipoprotein cholesterol; PA = physical activity; PE = preeclampsia; PPD = postpartum depression; PTB = preterm birth; SBP = systolic blood pressure. ↑ = higher with exercise, ↔ = similar with PA/exercise, ↓ = decreased with PA or exercise.

### Traditional CVD risk factor determinants

#### Blood pressure

According to a recent meta-analysis, exercise during pregnancy can lower systolic and diastolic BP by about 2-3 mm Hg (systolic: −3.19, 95% CI: −5.13 to −1.25 mm Hg; diastolic: −2.14, 95% CI: −4.26 to −0.03 mm Hg).^21^ Systolic BP was 7.5 mm Hg (95% CI: 1.5-12.6, *P* = 0.013) lower following a 12-week uphill walking exercise intervention in normotensive participants.^38^ This finding is important; even moderately elevated prenatal BP is associated with higher risk of hypertension years after delivery.^39^

Pregnant individuals at high risk of HDP had a smaller increase in systolic and diastolic BP from week 14 to 34 of pregnancy with a walking intervention; the mean systolic BP increase was 1.81 ± 2.40 mm Hg in walking group vs 9.87 ± 2.87 mm Hg in control group (*P* = 0.03).^40^ Regular PA during pregnancy appears to help prevent HDP and assist with management and severity in those diagnosed.^7^^,^^40^ Benefits are seen in individuals who do and do not exercise prior to becoming pregnant.^13^ PA prior to and during early pregnancy were associated with reductions in preeclampsia risk between 20% and 35%, with the greatest reductions seen in those engaged in PA prior to pregnancy (relative risk: 0.65; 95% CI: 0.47-0.89).^41^ Authors hypothesized that PA before and during early pregnancy could protect against hypertensive APOs due to improved placentation.^41^ The greatest benefits of PA were observed with 5 to 6 hours per week of exercise.^41^ This volume of PA is much greater than current guidelines for nonpregnant and pregnant adults.^6^

Less is known regarding the influence of prenatal PA or exercise on BP after delivery. An observational study reported that greater moderate-to-vigorous physical activity throughout pregnancy was linked to better BP in the first weeks after delivery.^42^ A sub-analysis of a trial that began in early pregnancy and focused on preventing excessive gestational weight gain (GWG) by counseling participants to eat healthfully and be more active found no effect of the intervention on BP patterns from early pregnancy to 12 months postpartum.^43^

#### Glucose

Performing high amounts of exercise (760 MET·min/wk) in early pregnancy–mid-second trimester was associated with a decrease of 3.9 mg/dL in glucose (95% CI: −7.4 to −0.5 mg/dL) compared with low exercise levels.^22^ According to a systematic review investigating associations between prenatal exercise and blood glucose, women with diabetes can improve fasting blood glucose following chronic exercise training (mean difference: −2.76 mg/dL, 95% CI: −3.18 to −2.34 mg/dL), an effect not observed in women free from diabetes (mean difference: −0.05 mg/dL, 95% CI: −0.16 to 0.05 mg/dL), although quality of evidence was “low.”^44^

GDM is a carbohydrate intolerance first observed during pregnancy and is associated with greater risk of hypertensive APOs, including gestational hypertension and preeclampsia.^45^ GDM is associated with higher lifetime risk of developing cardiometabolic diseases (CMDs).^46^ PA is linked to a lower risk of GDM, and National PA Guidelines recommend PA/exercise to help prevent GDM.^6^^,^^10^ Once GDM is diagnosed, lifestyle modification, including moderate exercise, is recommended for first-line management.^45^ Lifestyle modification, including increasing PA, was one of the few strategies identified for combating excess CMD risk after GDM in a recent review.^47^

#### Body mass index

Increases in body mass index do not always completely reverse after delivery, predisposing individuals to risk of obesity.^48^ The Institute of Medicine sets pregnancy weight gain goals based on pre-pregnancy body mass index to optimize pregnancy outcomes,^49^ yet only 30% to 40% of women gain the recommended amount of weight.^50^ Excessive GWG and postpartum weight retention are associated with excess CMD risk in individuals who did and did not have GDM.^51^^,^^52^ Historically, there was concern that prenatal exercise increases risk of inadequate GWG; these fears have been refuted.^13^

National PA Guidelines and others concluded that prenatal/perinatal exercise lowered odds of excessive GWG and promoted better postpartum weight, especially when combined with healthy diet.^10^^,^^23^ ACOG recommends obesity management prior to pregnancy, as even small weight reductions may improve pregnancy outcomes.^53^ A randomized controlled trial that began in early pregnancy among individuals who were overweight or obese prior to pregnancy counseled participants to eat healthfully and be more active demonstrated mildly favorable or null effects of the intervention on GWG depending on race and weight at study entry.^54^ However, the same study found postpartum weight outcomes were better in the intervention group: intervention participants retained 3.6 kg less weight (95% CI: −5.5 to −1.8 kg) at 6 months postpartum and 2.4 kg less weight (95% CI: −4.3 to −0.5 kg) at 12 months postpartum. At 6 months postpartum, overweight-intervention participants retained 4.1 kg less weight (95% CI: −6.7 to −1.5 kg), and obese intervention participants retained 3.3 kg less weight (95% CI: −5.8 to −0.7 kg) than control groups of the same weight status.^55^

#### Smoking

Pregnancy has been identified as an ideal time to intervene on smoking behaviors as pregnant women tend to have increased access to healthcare and may be motivated by fetal well-being.^56^ Health behaviors often cluster; PA interventions may have off-target effects on smoking. However, lifestyle interventions have not demonstrated consistent benefits on smoking cessation or relapse prevention in postpartum women.^57^ Studies that utilized a PA intervention as a smoking-cessation treatment have not been effective, although better PA quantification is needed.^58^

#### Diet

PA + nutrition interventions are recommended to combat obesity around the perinatal period.^53^ These combined interventions have had mixed effects on other pregnancy-specific CVD risk factors: GDM, excessive GWG, BP, APOs, and postpartum weight retention.^43^

#### Lipids

During pregnancy, cholesterol levels can increase by 50% to 70%.^59^ High maternal cholesterol levels during pregnancy may be associated with risk of CVD in offspring.^60^ Maternal dyslipidemia is associated with multiple APOs.^61^ Maternal lipid levels were improved with prenatal exercise (low-density lipoprotein levels mean change: −8 mg/dL, 95% CI: −3 to −29 mg/dL, *P* < 0.001) and triglycerides (mean change: −6 mg/dL, 95% CI: −1 to −11 mg/dL, *P* = 0.03)^22^^,^^26^^,^^27^ with nonsignificant improvements or no benefits seen with postpartum exercise in lactating women.^62^^,^^63^

### Novel CVD risk factor determinants

#### Sleep

Poor sleep is an independent risk factor for CVD.^64^ Pregnancy has been associated with sleep disturbances and lower sleep quality.^65^ Greater sleep disturbances were directly associated with higher odds of preeclampsia (odds ratio [OR]: 2.80, 95% CI: 2.38-3.30), HDP (OR: 1.74, 95% CI: 1.54-1.97), GDM (OR: 1.59, 95% CI: 1.45-1.76), preterm birth (OR: 1.38, 95% CI: 1.26-1.51), and stillbirth (OR: 1.25, 95% CI: 1.08-1.45**)**.^66^ Exercise improves perinatal sleep, especially when initiated during early pregnancy,^28^ with improvements in sleep quality detected in a meta-analysis (OR: 6.21, 95% CI: 2.02-19.11, *P* = 0.001).^67^

#### Depression and anxiety

Depression and anxiety are strong CVD risk factors, especially for women.^68^ Depression and anxiety affect about 12% of pregnant and postpartum women.^69^^,^^70^ Exercise/PA can lower postpartum depression symptoms.^6^^,^^71^

#### Placental characteristics

Inadequate placental formation and vascularization is a seminal event in the pathogenesis of multiple APOs.^72^ Pan-vascular adaptations to exercise, including reduced inflammation and improved angiogenesis, may benefit placental development.^73^ Maternal exercise during pregnancy may influence placentation or placental characteristics.^73^ Pathological features, such as malperfusion lesions, have been associated with APOs and subclinical CVD after delivery.^74^^,^^75^

Better placental growth, volume, and function have been described in women who began or continued vigorous exercise (running) during pregnancy vs women who did not.^76^ A recent study found no association of moderate-to-vigorous physical activity with placental malperfusion lesions, but results were limited by the convenience sample that included only 50 individuals with medically indicated placental pathology examinations.^42^ Soluble FMS-like tyrosine-kinase-1 is a placenta-derived angiogenic inhibitor that is elevated in preeclampsia.^77^ Individuals in a national, diverse sample who were meeting PA guidelines had lower soluble FMS-like tyrosine-kinase-1 levels in the first trimester (846.3 pg/mL, 95% CI: 821.6-871.8) vs those who did not meet PA guidelines (893.0 pg/mL, 95% CI: 864.5-922.5, *P* < 0.017), potentially indicating a role for the placenta in the utility of PA for combating preeclampsia.^78^

#### Lactation

Lactation (dichotomous or dose-dependent) leads to both short- and long-term CMD risk-factor-determinant improvements, like insulin sensitivity, hypertension, lipid profiles, and glucose levels; is associated with 25% to 47% lower diabetes risk through late middle age; and is associated with lower lifetime CVD risk (HR: 0.89, 95% CI: 0.83-0.95).^79^^,^^80^ Individuals affected by APOs may be less likely to lactate due to multiple biological and logistical factors, such as delayed lactogenesis or infant neonatal intensive care unit admission.^81^^,^^82^ With appropriate caloric intake, breastmilk volume and composition and infant growth are not influenced by moderate exercise.^37^ A recent national cohort study found that Black and White parous individuals who both lactated for ≥3 months during reproductive years and performed above average amounts of PA throughout adulthood had lower CMD risk scores in late middle age, suggesting joint (and not overlapping) effects of lactation and PA in adulthood.^83^

#### Arterial stiffness

Arterial stiffness is independently associated with CVD in people with and without hypertension.^84^ High arterial stiffness may be associated with greater risk of preeclampsia.^85^ Increases in arterial stiffness during pregnancy tend to normalize 7 weeks postpartum in most cases but may continue following delivery in women with preeclampsia, enhancing CVD risk later in life.^86^ A recent study concluded that aerobic exercise initiated after the first trimester in low-risk pregnancies does not appear to influence pregnancy-induced vascular adaptations, including arterial stiffness.^33^

#### Endothelial function

Endothelial function is enhanced during pregnancy, perhaps due to greater shear stress in arteries and higher circulating blood volume.^87^ Fetal growth restriction and HDP are often characterized by endothelial dysfunction.^87^ Regular exercise training can improve endothelial function in healthy pregnant individuals.^34^

#### Autonomic balance

Lower baroreflex sensitivity may be associated with greater risk of HDP.^88^ Higher than expected increases in resting heart rate may be associated with greater risk of HDP.^88^ Lower heart rate variability may be an important risk marker for HDP, suggesting sympathovagal imbalance may play a role in the etiology of HDP.^88^

A prospective study of 139 women reported higher beat-to-beat BP variability in every trimester in individuals who developed preeclampsia (systolic: first: 4.8 ± 1.3 vs 3.7 ± 1.2 mm Hg, *P* = 0.001; second: 5.1 ± 1.8 vs 3.8 ± 1.1 mm Hg, *P* = 0.02; third: 5.2 ± 0.8 vs 4.0 ± 1.1 mm Hg, *P* = 0.002; diastolic: first: 3.1 ± 0.9 vs 2.5 ± 0.7 mm Hg, *P* = 0.02; second: 3.0 ± 0.8 vs 2.5 ± 0.5 mm Hg, *P* = 0.007; third: 3.5 ± 1.3 vs 2.5 ± 0.8 mm Hg, *P* = 0.04).^35^ Elevated first trimester systolic beat-to-beat BP variability was associated with preeclampsia after statistical adjustment, OR: 1.94, 95% CI: 1.27-2.99.^35^ Sympathetic activity during pregnancy was improved in a recent, small trial of pregnant individuals who began aerobic exercise.^36^

#### Inflammation

In individuals who go on to develop some APOs, higher inflammation may be detectable early in pregnancy yet do not appear to pre-date pregnancy.^89^^,^^90^ C-reactive protein was lower in active (n = 16) vs inactive (n = 16) women with obesity (inactive: 9.1 ± 4.0 mg/L vs active: 6.3 ± 2.5 mg/L, *P* = 0.02).^30^ Women at risk of GDM enrolled in an exercise intervention (n = 84) or control group (n = 87) who maintained their exercise had a decrease in CRP while those who reduced their exercise had an increase in CRP (decreased PA: +0.09 mg/dL, 95% CI: −0.14 to 0.33; maintained PA: −0.08 mg/dL, 95% CI: −0.23 to 0.08, *P* < 0.05).^31^ Light PA was negatively and significantly correlated with maternal C-reactive protein (light PA: r = −0.40, *P* = 0.01, moderate PA: r = −0.18, *P* = 0.21).^32^

### Future directions to improve understanding of perinatal exercise and CVD risk

Areas in need of investigation are described in Table 4. Participation in perinatal exercise or PA is low. Effective, low-cost, perinatal exercise/PA promotion strategies are needed. Although entering pregnancy with an established exercise routine would be ideal, many US pregnancies are unplanned.^92^ Just-in-time interventions supporting the adoption and upkeep of exercise programs immediately before, during, and after pregnancy are critical. Given the role of social determinants of health in CVD risk and PA/exercise participation, perinatal exercise strategies must be tailored and adapted for individuals across a broad range of social and economic strata.

**Table 4:** Areas in Need of Research

Gaps in Research Informing Recommendations	Significance and Suggestions for Future Studies
Timing and dosing strategies	Optimal timing and dosing of the PA or exercise (pre-pregnancy, mid-pregnancy, or postpartum) are unknown. Some association studies concluded that prepregnancy or early pregnancy exercise/PA is especially helpful.^41^ Specific guidelines for return to exercise and fitness progression postpartum in athletes are lacking.
Resistance training exercise programs	Data are sparse regarding the use of perinatal resistance training on CVD risk. Resistance training alone can improve multiple CVD risk factors in the general population^14^ and might be more feasible for pregnant individuals, especially in late pregnancy or early postpartum when aerobic exercise might be less comfortable.
Gaps in research regarding relationships with perinatal exercise	
Placental features	Whether perinatal exercise influences placental formation or function is not well defined. The use of more sensitive indicators of placental function and large, unbiased samples might better identify the optimal dose and timing of exercise/PA for placental health.
Lactation	Recent evidence suggests joint rather than overlapping effects of PA/exercise and lactation on cardiometabolic disease (CMD) risk.^83^ Whether PA must be performed before, during, or after lactation to mitigate later life cardiometabolic risk is unknown.
Arterial stiffness	Arterial stiffness is independently associated with CVD.^84^ Data regarding associations of perinatal exercise with arterial stiffness during and after pregnancy are extremely limited.
Sedentary behavior	Sedentary behavior is a CVD risk factor. Preliminary data reveled that high sedentary behavior is a risk factor for APOs.^91^ Detailed recommendations regarding total amounts and consideration of sedentary behavior volume in relation to PA volume do not exist.

## Conclusions

PA and exercise are recommended for pregnant and postpartum individuals without contraindications, have beneficial effects on CVD risk during and after pregnancy, and do not adversely influence infant growth or breastmilk (Central Illustration).^10^^,^^93^ Granular levels of information regarding optimal types, timing, doses, progression, and adoption and adherence strategies are sparser than those in the general nonpregnant population.^6^ More nuanced guidelines for prescribing and monitoring PA in individuals entering pregnancy or postpartum with CVD/CMD should be developed. Overlapping or interactive effects of exercise/PA with perinatal-specific factors linked to long-term CVD/CMD risk, such as placental development and lactation, need more investigation. Trials utilizing behavior change strategies and technologies should be tested in this population.

**Central Illustration:**
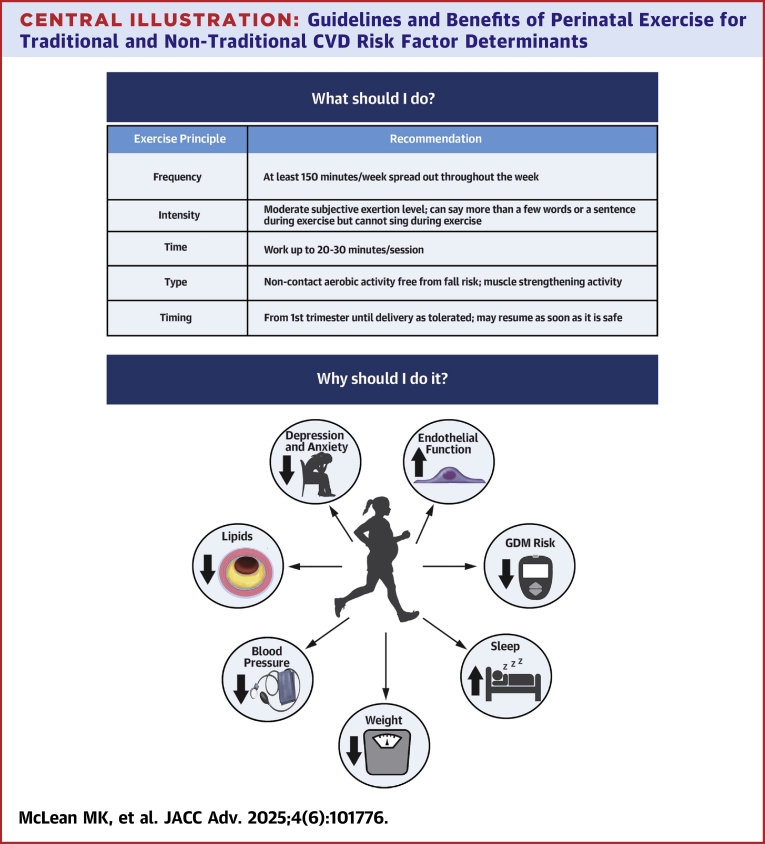
**Guidelines and Benefits of Perinatal Exercise for Traditional and Non-Traditional CVD Risk Factor Determinants** Created with BioRender.

## Funding support and author disclosures

The authors have reported that they have no relationships relevant to the contents of this paper to disclose.
