# Supplementary material for: Perinatal Exercise and Cardiovascular Disease Risk
Source: JACC Adv. 2025 May 12;4(6):101776. doi: 10.1016/j.jacadv.2025.101776 (PMC12141898; doi:10.1016/j.jacadv.2025.101776)
Supplement: Supplemental data [file mmc1.docx]

**SUPPLEMENTAL APPENDIX**

**Guidelines for Specific Subgroups of Pregnant Individuals**

ACOG guidelines state that healthy lifestyle modification, including PA and a healthy diet, should be encouraged for pregnant individuals with obesity. Exercise should initially be low in intensity and short in duration and increased as tolerated. Exercise in individuals with obesity can lead to modestly lower gestational weight gain (GWG) and lower incidences of preterm birth and GDM.^1^

One third of the global guidelines for exercise during pregnancy do not include recommendations for individuals who exercised regularly prior to becoming pregnant.^2^ Recommendations that do include these individuals state that those who were active or highly active prior to pregnancy can continue these activities during the pregnancy and postpartum periods, but can discuss adjustments in PA with their obstetrician.^1^ More research is needed regarding the safety of vigorous exercise performed early in pregnancy, though it seems to be safe in late pregnancy.^1^ There is a paucity of evidence assessing the risks and benefits of exercise among pregnant elite sportswomen, defined as an athlete training year-round with years of experience competing successfully against other elite performers,^3^ with more information available for recreationally active individuals as compared to competitive athletes.

**Recommendation gaps and research directions to inform guidelines**

*Expanding evidence on special populations.*

Although the ACOG exercise/PA guidelines include a special populations section, only considerations for athletes and overweight and obese individuals are specifically included in the PA guidelines paper.^1^ Recommendations for patients with known or de novo CVD (including congenital heart disease), chronic kidney disease (CKD), diabetes, psychiatric illness, or other chronic conditions known to enhance CVD risk are not specifically mentioned in the PA guideline statements, though exercise or PA are briefly mentioned in other ACOG guidelines for perinatal management of some chronic conditions. ACOG’s guidelines for management of chronic hypertension during pregnancy include lifestyle modifications.^4^ The guidelines for management of diabetes includes diet, exercise, and pharmacological therapies.^5^ A practice bulletin recommends a pre-pregnancy exercise stress test to estimate risk of cardiovascular complications of pregnancy in individuals with known heart disease.^6^ PA is mentioned in this bulletin to help support weight loss after delivery, though timing or dosing of PA is not discussed.^6^

*Uptake of PA guidelines.*

Strategies to promote PA or exercise uptake and adherence are briefly discussed in the ACOG guidelines,^1^ and patients are more likely to participate in PA if it is recommended by their physician.^7^ Motivational counseling is recommended for adoption of an exercise program.^1^ The Five A’s (Ask, Advise, Assess, Assist, and Arrange) have been successfully implemented for lifestyle behavior modification counseling.^1^ This method includes asking the individual about their current habits, providing clear advice, assessing the individual’s readiness to change, providing appropriate materials, and arranging for follow-up with professionals who can support their behavior change.^1^ The ACOG guidelines also recommend an individualized exercise program based on general population guidelines, with considerations made for medical history, prior exercise level, environmental conditions, and hydration status.^1^

*Timing of postpartum return to training for athletic individuals.*

More data supporting definitive return-to-sport guidelines are needed for elite and recreational athletes. A recent consensus statement on returning to running suggested that a minimum 3-week rest period should precede a gradual and individualized return to running after delivery for all runners who gave birth.^8^ Elite athletes are competing during childbearing years and returning to sport following childbirth more frequently than ever,^9^ necessitating more specific recommendations for this subpopulation. Systematic reviews investigating qualitative experiences of elite athletes during their pregnancies and postpartum return to sport found elite athletes reported both internal and external barriers while navigating pregnancy, further necessitating the development of best-practice guidelines for pre-pregnancy participation in elite sports.^9^ Elite athletes return to participation in exercise earlier than the recreationally active population, yet the earlier return comes with an increased risk of injury.^10,11^ Interestingly, 56% of world-class runners who returned to sport after pregnancy improved their marathon times suggesting roughly equivocal effects on endurance performance.^12^

Establishing return to sport guidelines may enhance both clinical and athletic performance outcomes. To support the return to sport or strenuous exercise, it is important to educate fitness and perinatal professionals on safe and effective training programs for clients of all stages of pre-pregnancy fitness. Training and certifying coaching entities should ensure the curriculum is aimed at training nonathletes/novice exercisers as well as elite and recreational athletes through pregnancy and postpartum with an emphasis on healthy pregnancies/births, CVD risk mitigation, and athletic performance when relevant.

1. Syed H, Slayman T, Thoma KD. ACOG committee opinion no. 804: Physical activity and exercise during pregnancy and the postpartum period. *Obstetrics & Gynecology*. 2021;137:375-376.

2. Hayman M, Brown WJ, Brinson A, Budzynski-Seymour E, Bruce T, Evenson KR. Public health guidelines for physical activity during pregnancy from around the world: a scoping review. *British Journal of Sports Medicine*. 2023.

3. Pivarnik JM, Szymanski LM, Conway MR. The elite athlete and strenuous exercise in pregnancy. *Clinical Obstetrics and Gynecology*. 2016;59:613-619.

4. American College of Obstetricians and G. ACOG Practice Bulletin No. 203: chronic hypertension in pregnancy. *Obstetrics and gynecology*. 2019;133:e26-e50.

5. American College of Obstetricians and G. ACOG practice bulletin no. 201: pregestational diabetes mellitus. *Obstetrics and gynecology*. 2018;132:e228-e248.

6. ACOG Practice Bulletin No. 212 Summary: Pregnancy and Heart Disease. *Obstet Gynecol*. 2019;133:1067-1072. doi: 10.1097/AOG.0000000000003244

7. Nawaz H, Adams ML, Katz DL. Physician—patient interactions regarding diet, exercise, and smoking. *Preventive Medicine*. 2000;31:652-657.

8. Christopher SM, Donnelly G, Brockwell E, Bo K, Davenport MH, De Vivo M, Dufour S, Forner L, Mills H, Moore IS, et al. Clinical and exercise professional opinion of return-to-running readiness after childbirth: an international Delphi study and consensus statement. *Br J Sports Med*. 2024;58:299-312. doi: 10.1136/bjsports-2023-107489

9. Davenport MH, Nesdoly A, Ray L, Thornton JS, Khurana R, McHugh T-LF. Pushing for change: a qualitative study of the experiences of elite athletes during pregnancy. *British Journal of Sports Medicine*. 2022;56:452-457.

10. Nose-Ogura S, Yoshino O, Nakamura-Kamoto H, Kanatani M, Harada M, Hiraike O, Saito S, Fujii T, Osuga Y. Medical issues for female athletes returning to competition after childbirth. *The Physician and Sportsmedicine*. 2023:1-7.

11. L’Heveder A, Chan M, Mitra A, Kasaven L, Saso S, Prior T, Pollock N, Dooley M, Joash K, Jones BP. Sports Obstetrics: Implications of Pregnancy in Elite Sportswomen, a Narrative Review. *Journal of Clinical Medicine*. 2022;11:4977.

12. Darroch F, Schneeberg A, Brodie R, Ferraro ZM, Wykes D, Hira S, Giles AR, Adamo KB, Stellingwerff T. Effect of Pregnancy in 42 Elite to World-Class Runners on Training and Performance Outcomes. *Medicine and Science in Sports and Exercise*. 2022;55:93-100.
